# *Poria cocos* Polysaccharide Fraction PCP-II Enhances Humoral and Cellular Responses to a SARS-CoV-2 RBD Subunit Vaccine in Mice

**DOI:** 10.3390/vaccines14050389

**Published:** 2026-04-27

**Authors:** Mao Zhou, Jing Liu, Xiaotuan Zhang, Feihu Yan, Yuan Wu, Cheng Huang, Dan Xie, Bin Liu

**Affiliations:** 1 Department of Clinical Laboratory, Second Affiliated Hospital, Hengyang Medical School, University of South China, Hengyang 421001, China; 2 Disinfection Supply Center, Second Affiliated Hospital, Hengyang Medical School, University of South China, Hengyang 421001, China; 3 Key Laboratory of Jilin Province for Zoonosis Prevention and Control, Changchun Veterinary Research Institute, Chinese Academy of Agricultural Sciences, State Key Laboratory of Pathogen and Biosecurity, Changchun 130122, China; 4 Department of Clinical Laboratory, Hengnan County People’s Hospital, Hengyang 421100, China

**Keywords:** SARS-CoV-2, receptor-binding domain (RBD), polysaccharide adjuvant, traditional Chinese medicine polysaccharides, *Poria cocos*, PCP-II

## Abstract

Background: The emergence of SARS-CoV-2 variants necessitates the development of effective adjuvants to enhance subunit vaccine immunogenicity. Safe adjuvants are essential to enhance the immunogenicity of SARS-CoV-2 receptor-binding domain (RBD) subunit vaccines. Traditional Chinese medicine polysaccharides are attractive candidates due to their immunomodulatory properties. Methods: Female BALB/c mice (6–8 weeks) were immunized on days 0, 7, and 21 with an RBD protein (20 μg) alone or formulated with *Poria cocos* polysaccharide fraction PCP-I or PCP-II (200 μg), *Isatis indigotica* polysaccharide, or aluminum adjuvant; PBS served as a control. RBD-specific total IgG and subclasses were quantified by ELISA on day 7 after the third immunization. Neutralizing antibody titers were measured by a pseudovirus assay on days 14, 28, and 56 after the first immunization. Splenic CD19^+^ B cells were analyzed by flow cytometry, and antigen-stimulated IFN-γ and IL-4 spot-forming cells were quantified by ELISpot. Results: PCP-II significantly increased RBD-specific total IgG and IgG1 compared with RBD alone and other formulations, whereas IgG2a and IgG2b remained unchanged. Both PCP-I and PCP-II increased neutralizing titers versus RBD alone, and PCP-II showed an earlier and sustained increase in neutralizing responses through day 56. PCP-II showed a non-significant increase in splenic CD19^+^ B cell frequency. PCP-I and PCP-II markedly increased IFN-γ-secreting splenocytes without increasing IL-4, indicating enhanced antigen-specific cellular responses. Conclusion: In this comparative evaluation of traditional Chinese medicine polysaccharide candidates in a SARS-CoV-2 RBD subunit vaccine model, PCP-II showed the most prominent adjuvant activity. PCP-II enhanced antigen-specific humoral immunogenicity, improved neutralizing antibody responses, and was associated with increased IFN-γ-related cellular responses, supporting its potential as a candidate polysaccharide adjuvant for protein subunit vaccines.

## 1. Introduction

Coronavirus disease 2019 (COVID-19), caused by severe acute respiratory syndrome coronavirus 2 (SARS-CoV-2), has resulted in substantial mortality and major socioeconomic losses since its emergence and remains a serious global public health challenge. In recent years, continued viral evolution has produced variants with enhanced transmissibility and immune evasion compared with early strains, and resurgences of infections have been observed in multiple regions worldwide [1]. Vaccination remains the most effective strategy for preventing COVID-19 and reducing severe disease caused by SARS-CoV-2 [2]. Most neutralizing antibodies in convalescent sera target the receptor-binding domain (RBD) of the spike protein, making RBD a key antigen for vaccine development [3]. However, protein subunit vaccines based on RBD generally require effective adjuvants to enhance immunogenicity and improve the durability of immune responses. Beyond viral evolution, the relatively short durability of immunity induced by many current vaccines, weaker responses in specific populations (e.g., older adults and immunocompromised individuals), and the need to maintain population-level protection continue to drive innovation in vaccine research.

Traditional Chinese medicine (TCM) polysaccharides are natural high-molecular-weight polymers that interact with receptors on multiple immune cell types, activate downstream signaling pathways, promote cytokine secretion, and enhance immune responses. They have therefore been widely explored as vaccine adjuvants [4,5,6,7]. Compared with conventional adjuvants such as aluminum salts, TCM polysaccharides have attracted increasing attention due to their immunomodulatory activity, biocompatibility, biodegradability, low toxicity, and favorable safety profiles [8]. For example, *ginseng* polysaccharide significantly enhances both humoral and cellular immune responses when used as an adjuvant for an RBD-based SARS-CoV-2 subunit vaccine in mice [9]. *Astragalus* polysaccharide also shows robust adjuvant activity for influenza and SARS-CoV-2 RBD subunit vaccines and can improve survival in experimental settings [10]. Collectively, these findings suggest that TCM polysaccharides are promising adjuvant candidates that may improve the immunogenicity of subunit vaccines, with potential utility in responding to global public health emergencies such as COVID-19.

*Isatis indigotica* (Banlangen) and *Poria cocos* are traditional TCM materials that have been used in the treatment of respiratory infections such as influenza and SARS [11,12,13]. Previous studies show that Banlangen polysaccharide used as an adjuvant for an H1N1 split vaccine and an inactivated rabies vaccine markedly increases antigen-specific antibody titers and, overall, can outperform aluminum adjuvants [7,14]. Two polysaccharide fractions isolated from *Poria cocos*, designated PCP-I and PCP-II, display adjuvant activity for inactivated influenza vaccines, hepatitis B surface antigen (HBsAg), and inactivated rabies vaccines. These fractions can substantially enhance both humoral and cellular immune responses and improve survival following viral challenge [15]. However, the immunostimulatory effects of Banlangen polysaccharide and *Poria cocos* polysaccharides (PCP-I and PCP-II) as adjuvants for SARS-CoV-2 vaccines have not been reported.

Therefore, in the present study, we evaluated the adjuvant effects of Banlangen polysaccharide and *Poria cocos* polysaccharides I and II in a SARS-CoV-2 RBD protein subunit vaccine model, with the aim of assessing their potential as natural adjuvant candidates for the development of more effective subunit vaccines.

## 2. Methods

### 2.1. Polysaccharides, Antigen, and Animals

Banlangen polysaccharide, *Poria cocos* polysaccharides PCP-I and PCP-II I, and the SARS-CoV-2 RBD protein were provided by Prof. Feihu Yan. at the Laboratory of Animal Virology and Special Animal Epidemics, Changchun Veterinary Research Institute, Chinese Academy of Agricultural Sciences. The recombinant RBD protein was expressed in a prokaryotic expression system (*Escherichia coli*) and purified prior to use. Female BALB/c mice (6–8 weeks old; 18–20 g) were purchased from Jiangsu Jicui Yaokang Co., Ltd. (Nanjing, China).

All animal experiments were approved by the Medical Research Ethics Committee at The Second Affiliated Hospital, University of South China. Housing and experimental procedures complied with the national standards for laboratory animals in China (GB 14925-2001) [16]. This study followed the Helsinki Declaration of 1975.

A total of 72 mice were randomly assigned to six groups (*n* = 12 per group): PBS control, SARS-CoV-2 RBD, PCP-I + RBD, PCP-II + RBD, *Isatis indigotica* polysaccharide (IIP) + RBD, and aluminum adjuvant (Al) + RBD. All formulations were administered by subcutaneous injection in the dorsal interscapular region, with a final injection volume of 50 μL per mouse. The dose of the RBD antigen was 20 μg per mouse, and the dose of each polysaccharide adjuvant was 200 μg per mouse. In the co-immunization groups, the adjuvant was thoroughly mixed with the RBD antigen before injection, while mice in the control group received an equal volume of PBS. Immunizations were carried out on days 0, 7, and 21. Serum samples were collected 1 week after the second and third immunizations.

### 2.2. ELISA for Total IgG and IgG Subclasses

Seven mice were randomly selected from each group. On day 7 after the third immunization (day 28), whole blood (1–2 mL) was collected by retro-orbital bleeding into 1.5 mL tubes. Samples were incubated at 37 °C for 2 h and centrifuged at 3000 rpm for 10 min. Serum was collected, heat-inactivated at 56 °C for 30 min, and stored at −20 °C until analysis.

For ELISA, 96-well plates were coated overnight at 4 °C with the RBD protein (100 ng per well in 100 μL). Plates were washed four times with 1× PBST (5 min each; 600 rpm) and blotted dry. Wells were blocked with 200 μL 1× BSA at 37 °C for 4.5 h. Serially diluted mouse sera (150 μL per well) were added and incubated at 37 °C for 2 h. Plates were washed four times with PBST and incubated for 1 h at room temperature with HRP-conjugated goat anti-mouse IgG, IgG1, IgG2a, or IgG2b (Southern Biotechnology Associates, Inc. Birmingham, AL, USA; 1:1000 in PBST; 200 μL per well). After the plates were washed five times, TMB substrate (100 μL/well) was added, and the reaction was allowed to develop in the dark for 5–15 minThe reaction was stopped with 50 μL stop solution, and absorbance at 450 nm (A450) was measured using a microplate reader.

### 2.3. Virus Neutralization Assay

Neutralizing antibody responses were evaluated using a recombinant SARS-CoV-2 Wuhan-01 strain expressing enhanced green fluorescent protein (eGFP), which was provided by Prof. Feihu Yan at the Laboratory of Animal Virology and Special Animal Epidemics, Changchun Veterinary Research Institute, Chinese Academy of Agricultural Sciences.

Blood samples were collected at 7 days after the second immunization, 7 days after the third immunization, and 35 days after the third immunization (corresponding to days 14, 28, and 56). On day 14, blood samples were collected from seven randomly selected mice in each group. On day 28, neutralizing antibody titers were analyzed using the same blood samples described in Section 2.2. On day 56, the remaining 7 mice—those left after spleen collection—were sampled. Whole blood was obtained via retro-orbital bleeding as described in Section 2.2 and transferred into 1.5 mL centrifuge tubes. The samples were incubated at 37 °C for 2 h and then centrifuged at 3000 rpm for 10 min to collect the serum. Mouse sera were heat-inactivated at 56 °C for 30 min prior to use. Serial two-fold dilutions of sera were prepared in DMEM starting from an initial dilution of 1:4 in 96-well plates. Each serum dilution was incubated with 100 TCID_50_ of the recombinant virus at 37 °C for 1 h to allow for antibody–virus interaction. Subsequently, Vero E6 cells were added to each well at a density of approximately 1 × 10^4^ cells per well, and the plates were incubated at 37 °C in a 5% CO_2_ atmosphere. Appropriate controls were included in each assay, including a cell-only control, a virus-only control, and a positive control serum (equine anti-SARS-CoV-2 serum provided by Prof. Feihu Yan at the Laboratory of Animal Virology and Special Animal Epidemics, Changchun Veterinary Research Institute, Chinese Academy of Agricultural Sciences).

After 48 h, infection was assessed by detecting eGFP fluorescence using a fluorescence microscope. The neutralizing antibody titer was defined as the highest serum dilution that completely inhibited detectable green fluorescence in infected cells.

### 2.4. Splenocyte Isolation

On day 7 after the third immunization (day 28), spleens were aseptically collected from five mice per group. Spleens were gently dissociated through a 70 μm cell strainer using a syringe plunger. Single-cell suspensions were prepared at 1 × 10^6^ cells/mL in RPMI 1640 supplemented with 10% fetal bovine serum (FBS). Cells were counted using a hemocytometer and adjusted to the desired density for subsequent assays.

### 2.5. Flow Cytometric Analysis of Splenic B Cells

Splenocytes were centrifuged at 4 °C (400× *g*, 5 min), and the supernatant was discarded. Red blood cells were lysed with ACK lysis buffer for 2–3 min and quenched with excess PBS containing 2% FBS. Cells were washed twice and resuspended in staining buffer (PBS + 2% FBS).

To block Fc receptor-mediated nonspecific binding, cells were incubated with anti-mouse CD16/32 antibody (BioLegend, San Diego, CA, USA) at 4 °C for 10 min. Cells were then stained with BV421-conjugated anti-CD19 antibody (BioLegend, San Diego, CA, USA) (1:100) at 4 °C for 30 min in the dark. After two washes, cells were resuspended in PBS and analyzed using a BD FACSCanto II flow cytometer (BD Biosciences, Milpitas, CA, USA). At least 100,000 events were acquired per sample. Data were analyzed using FlowJo software v10.8.

### 2.6. ELISpot for IFN-γ and IL-4

Antigen-specific IFN-γ- and IL-4-secreting cells were quantified using ELISpot kits (Mouse IFN-γ and IL-4 ELISpot kits; Mabtech AB, Stockholm, Sweden) according to the manufacturer’s instructions. Briefly, ELISpot plates were pre-washed with RPMI 1640 medium supplemented with antibiotics (200 μL per well) and incubated for 10 min. The plates were then emptied, and residual liquid was removed. Splenocytes were isolated from immunized mice and seeded at a density of 2.5 × 10^5^ cells per well in ELISpot plates. Cells were stimulated with a SARS-CoV-2 RBD protein (1 μg per well) and incubated at 37 °C for 24 h. For controls, positive control wells were stimulated with the kit-provided stimulant (CoA), while negative control wells received an equal volume of culture medium. The subsequent steps, including detection antibody incubation, enzyme conjugation, and substrate development, were performed strictly according to the manufacturer’s instructions. Finally, spot-forming cells (SFCs) were enumerated using an automated ELISpot reader (AID GmbH, Strassberg, Germany). All samples were tested in duplicate.

### 2.7. Statistical Analysis

Antibody levels, neutralizing titers, flow cytometry data, and ELISpot results were analyzed using a one-way analysis of variance (ANOVA) or *t*-tests as appropriate. All analyses were performed in GraphPad Prism 10.6. A *p* < 0.05 was considered statistically significant.

## 3. Results

### 3.1. PCP-II Increased RBD-Specific IgG and IgG1 Responses in Mice

**Figure 1:**
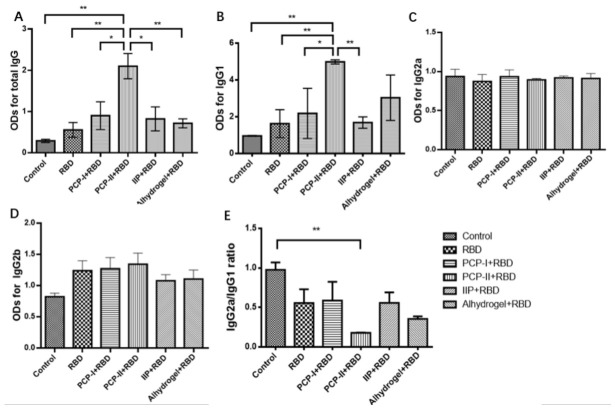
PCP-II enhances RBD-specific humoral immune responses in mice. Mice were immunized on days 0, 7, and 21. On day 7 after the third immunization (day 28), blood samples were collected from seven randomly selected mice per group. Antibody levels were measured by ELISA, and OD values at 450 nm (OD450) were recorded. (**A**) ODs for total IgG, (**B**) ODs for IgG1, (**C**) ODs for IgG2a, (**D**) ODs for IgG2b, and (**E**) the IgG2a/IgG1 ratio. Data are presented as the mean ± SD (*n* = 7). Statistical significance was determined by a one-way ANOVA followed by multiple comparisons. * *p* < 0.05, ** *p* < 0.01.

On day 7 after the third immunization, sera were collected and analyzed for antigen-specific total IgG and IgG subclasses (IgG1, IgG2a, IgG2b). As shown in Figure 1, the PCP-I + RBD, PCP-II + RBD, and IIP + RBD groups tended to show higher levels of total IgG and IgG1 compared with the RBD-only group, Al + RBD group, and PBS control group.

Notably, the PCP-II + RBD group exhibited significantly higher levels of total IgG than the PBS control, RBD-only, PCP-I + RBD, IIP + RBD, and Al + RBD groups (*p* < 0.05). Similarly, IgG1 levels in the PCP-II + RBD group were significantly higher than those in the PBS control, RBD-only, PCP-I + RBD, and IIP + RBD groups (*p* < 0.05), while no significant difference was observed compared with the Al + RBD group. In contrast, no significant differences were observed among groups for IgG2a or IgG2b. In addition, the IgG2a/IgG1 ratio was calculated as an indicator of antibody subclass distribution. The PCP-II + RBD group exhibited the lowest IgG2a/IgG1 ratio among all groups and was significantly lower than that of the PBS control group (*p* < 0.05), consistent with the elevated IgG1 response observed in this group.

These data indicate that PCP-II effectively enhances RBD-specific IgG and IgG1 responses, consistent with augmented humoral immunogenicity and an IgG1-dominant antibody response pattern in this model.

### 3.2. PCP-II Increased Neutralizing Antibody Titers and Accelerated Antibody Kinetics

**Figure 2:**
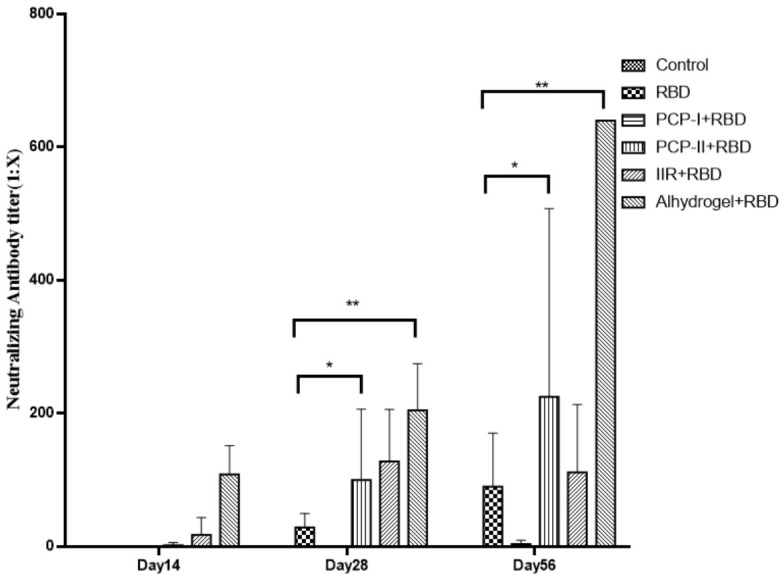
PCP-II enhances neutralizing antibody responses and accelerates antibody kinetics in mice. Neutralizing antibody titers were measured by a pseudovirus neutralization assay on days 14, 28, and 56 after the first immunization. Data are presented as the mean ± SD (*n* = 7). Statistical significance was determined by a one-way ANOVA followed by multiple comparisons. * *p* < 0.05, ** *p* < 0.01. PCP-II promoted earlier and sustained neutralizing antibody responses compared with the RBD-only group.

Neutralizing antibody titers were assessed by a pseudovirus neutralization assay on days 14, 28, and 56 after the first immunization. As shown in Figure 2, neutralizing antibodies were detectable as early as day 14 in the PCP-II + RBD group, whereas minimal responses were observed in the RBD-only group at this time point.

By day 28, both the PCP-I + RBD and PCP-II + RBD groups showed significantly higher neutralizing titers than the RBD-only group (*p* < 0.05). At day 56, neutralizing titers further increased in all adjuvanted groups, with the PCP-II + RBD group maintaining substantially higher levels than the RBD-only group (*p* < 0.05). The Al + RBD group tended to show the highest titers at later time points.

Notably, the PCP-II + RBD group displayed an earlier onset of antibody responses compared with the RBD-only group, and neutralizing titers continued to increase from day 14 to day 56, indicating that PCP-II enhanced the immunogenicity of the RBD vaccine and promoted an earlier and sustained neutralizing antibody response.

### 3.3. PCP-II Does Not Significantly Alter the Proportion of Splenic B Cells After RBD Stimulation

**Figure 3:**
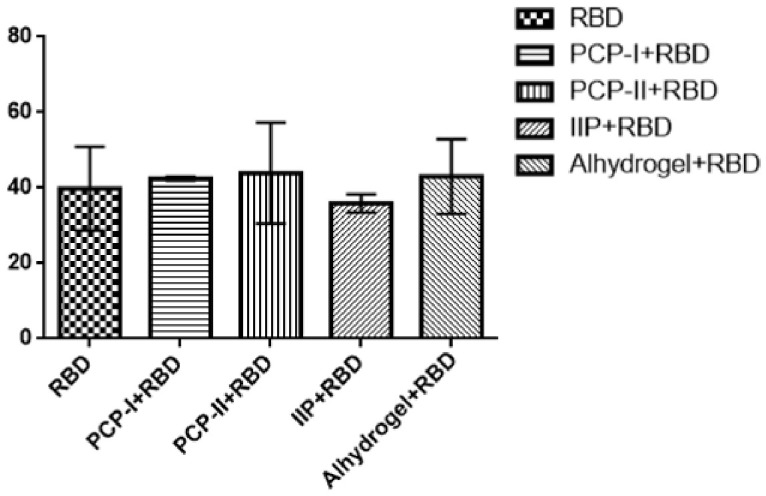
The effects of PCP-II on splenic B cell responses in mice. Splenic lymphocytes were isolated on day 7 after the third immunization, and the proportion of CD19^+^ B cells was analyzed by flow cytometry. Data are presented as the mean ± SD (*n* = 5). No significant differences were observed among groups (*p* > 0.05).

To assess whether *Poria cocos* polysaccharide adjuvants modulate humoral immune responses, splenic B cells were analyzed by flow cytometry. The proportions of CD19^+^ B cells were comparable among all groups. Although the PCP-II + RBD group showed a slightly higher mean value than some groups in Figure 3, these differences were not statistically significant (*p* > 0.05). These results suggest that PCP-II does not markedly affect the overall proportion of splenic B cells, and its adjuvant effect may not be the expansion of B cell populations.

### 3.4. PCP-II Enhances Antigen-Specific Cellular Immune Responses

**Figure 4:**
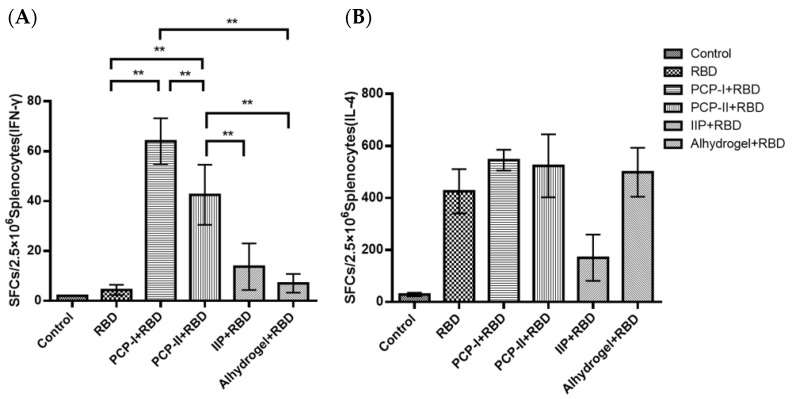
PCP-II enhances antigen-specific cellular immune responses in mice. Splenocytes were isolated on day 7 after the third immunization and stimulated with the SARS-CoV-2 RBD protein in vitro. Antigen-specific cellular responses were evaluated by ELISpot assays for (**A**) IFN-γ and (**B**) IL-4. Data are presented as spot-forming cells (SFCs) per 2.5 × 10^5^ splenocytes and shown as the mean ± SD (*n* = 5). Statistical significance was determined by a one-way ANOVA followed by multiple comparisons. ** *p* < 0.01. PCP-I and PCP-II markedly increased IFN-γ responses compared with other groups.

Following the confirmation of adjuvant-enhanced antibody responses, antigen-specific cellular immunity was assessed by ELISpot for IFN-γ and IL-4. As shown in Figure 4, the PCP-I + RBD and PCP-II + RBD groups exhibited significantly higher numbers of IFN-γ spot-forming cells than the other four groups (*p* < 0.001). In contrast, no clear differences in IL-4 spot-forming cells were observed among the four adjuvanted groups compared with the RBD-only group. These results indicate that PCP-I and PCP-II enhanced IFN-γ-associated antigen-responsive cellular responses under the present experimental conditions.

## 4. Discussion

The COVID-19 pandemic has transitioned from an acute global emergency into a phase characterized by long-term circulation with periodic rebounds. Continuous viral evolution and the emergence of immune-evasive variants have increased the demand for vaccines capable of inducing broader and more durable protection [17,18]. Among multiple vaccine platforms, subunit vaccines remain attractive for booster strategies because of their favorable safety profiles, stability, and mature manufacturing processes, making them particularly suitable for older adults and individuals with reduced immune function. However, subunit vaccines often require adjuvants to enhance immunogenicity—especially to elicit high levels of neutralizing antibodies and effective cellular immunity—which remains a key bottleneck limiting vaccine efficacy and durability [19].

Natural product-derived TCM polysaccharides have increasingly been investigated as vaccine adjuvants due to their biocompatibility, immunomodulatory activity, and relatively low systemic toxicity [4,20]. In this study, we used SARS-CoV-2 RBD as a model antigen and systematically evaluated the immunostimulatory effects of Banlangen polysaccharide and *Poria cocos* polysaccharides PCP-I and PCP-II as candidate adjuvants, aiming to explore their application potential in SARS-CoV-2 subunit vaccines. The novelty of the present study lies not in establishing a complete mechanism of PCP-II adjuvanticity but in providing a comparative evaluation of several traditional Chinese medicine polysaccharide candidates within the same SARS-CoV-2 RBD subunit vaccine model and identifying PCP-II as the fraction with the most prominent activity under the tested conditions. Our results indicate that *Poria cocos* polysaccharides, particularly PCP-II, exhibits promising adjuvant activity in enhancing vaccine-induced immune responses for RBD-based subunit vaccines by enhancing multiple aspects of vaccine-induced immune responses.

Antigen-specific IgG is a fundamental indicator of humoral immunogenicity for protein subunit vaccines [21]. We found that PCP-II significantly increased RBD-specific IgG and IgG1 levels in mice, indicating improved immunogenicity. In murine models, IgG1 is commonly the dominant subclass elicited by protein antigens and is often associated with enhanced B cell activation and antibody secretion [22]. The reduced IgG2a/IgG1 ratio further suggests a tendency toward an IgG1-dominant response, which is consistent with an IgG1-dominant humoral response. Thus, the elevation of IgG and IgG1 suggests that PCP-II promotes humoral immune priming in the murine RBD immunization model. A limitation of this study is that antibody avidity and affinity were not assessed. Therefore, although PCP-II enhanced antigen-specific IgG and neutralizing antibody responses, the quality and maturation of the induced antibodies remain to be further characterized.

Notably, in addition to increasing binding antibody levels, PCP-II also markedly improved neutralizing antibody titers in immunized mice. Neutralizing antibodies block viral entry mediated by spike–ACE2 interactions, and their levels correlate with vaccine-induced protective efficacy; therefore, neutralizing titers are widely used as functional correlates of protection for SARS-CoV-2 vaccines [23,24]. Compared with measurements of binding antibodies alone, pseudovirus neutralization assays more directly reflect the functional antiviral activity of vaccine-elicited antibodies [25,26]. The enhanced neutralizing titers induced by PCP-II suggest that this adjuvant may improve the magnitude, thereby increasing the protective potential of an RBD subunit vaccine. Furthermore, an earlier and sustained increase in neutralizing antibody titers observed in the PCP-II group suggests that PCP-II may accelerate the development of vaccine-induced antibody responses. For respiratory viruses such as SARS-CoV-2, faster antibody kinetics may provide practical public health advantages during outbreaks or periods of high transmission.

The spleen is a key organ for humoral immunity in mice; accordingly, we measured CD19^+^ splenic B cells to assess whether adjuvantation activated the humoral immune compartment. In the present study, the proportions of splenic B cells were comparable among groups, and no statistically significant differences were observed. While it should be noted that total splenic B cell frequency does not reflect antigen-specific responses and is largely maintained by homeostatic mechanisms. Taken together with the enhanced antigen-specific IgG and neutralizing antibody responses, these results suggest that the adjuvant activity of PCP-II is unlikely to be explained solely by changes in B cell frequency. Further studies are required to clarify its potential effects on B cell function and antibody maturation.

We further evaluated cellular immunity by ELISpot. Both PCP-I and PCP-II significantly increased IFN-γ-secreting splenocytes, indicating enhanced IFN-γ-associated cellular immune responses. IFN-γ plays a pivotal role in antiviral defense. In the context of respiratory virus vaccines, IFN-γ-associated responses have been discussed as potentially contributing to the reduced risk of Th2-biased vaccine-associated enhanced respiratory disease [27,28,29]. However, as the ELISpot assay does not distinguish the cellular sources of IFN-γ production, these responses may originate from multiple cell types, including CD4^+^ T cells, CD8^+^ T cells, and NK cells. Therefore, the current data should be interpreted as reflecting overall IFN-γ-associated cellular immune responses. Overall, PCP-II appears to improve RBD vaccine-induced immunity primarily through functional enhancement (antibody magnitude and neutralizing activity, IFN-γ-associated cellular responses) rather than by simply increasing immune cell abundance.

*Poria cocos* is the dried sclerotium of *Poria cocos* (Schw.) Wolf (Polyporaceae), and its polysaccharide constituents exhibit a variety of biological activities, including anti-tumor, anti-inflammatory, antioxidant, and immunomodulatory effects [30]. PCP-I and PCP-II refer to distinct polysaccharide fractions isolated from *Poria cocos*. Although interest in TCM polysaccharides as vaccine adjuvants has grown, reports on *Poria cocos* polysaccharides remain limited. Previous studies indicate that *Poria cocos* polysaccharides can enhance cellular immunity and induce Th1-associated responses, supporting their potential as Th1-type adjuvants [31]. PCP-I has been shown to improve the immunogenicity and safety of anthrax protective antigen [32]. PCP-II enhances antigen-specific antibody responses to H1N1 influenza vaccines, and HBsAg and has been reported to potentiate both humoral and cellular immunity [16]. In a rabies inactivated vaccine model, Zhang and colleagues found that PCP-II significantly increased virus-neutralizing antibody titers and demonstrated strong adjuvant effects under different immunization regimens compared with aluminum adjuvant [33].

In this study, the aluminum-adjuvanted group exhibited higher neutralizing antibody titers, which is consistent with the well-established ability of aluminum salts to preferentially enhance humoral immune responses. Aluminum salts are among the most widely used clinical adjuvants and effectively enhance antibody responses to protein antigens; however, they tend to favor Th2-biased immunity and may be less effective at inducing robust Th1 cellular responses and high-quality functional neutralizing antibodies [19,34]. In contrast, PCP-II increased RBD-specific IgG and IgG1 levels and significantly increased IFN-γ-secreting cell responses, suggesting that its immunological value may lie not in maximizing antibody magnitude alone but in promoting coordinated humoral and cellular immune responses. In addition, given the ongoing concerns regarding aluminum-containing formulations, the identification of effective non-aluminum adjuvants remains of practical interest. In this context, PCP-II may represent a promising non-aluminum adjuvant candidate capable of eliciting a robust immune response. Further studies are needed to clarify its underlying mechanisms and evaluate its broader applicability in vaccine platforms.

Despite these encouraging findings, from a translational perspective, natural polysaccharide adjuvants also present inherent challenges. Their structural complexity and heterogeneity may lead to batch-to-batch variability depending on source, extraction, and purification processes, which can affect reproducibility and standardization [35]. Moreover, their complex molecular composition makes mechanistic characterization more difficult compared with well-defined adjuvants [36]. Large-scale production, quality control, and regulatory standardization will therefore require further optimization.

In summary, our study demonstrates that the *Poria cocos* polysaccharide fraction PCP-II effectively enhances immune responses induced by a SARS-CoV-2 RBD protein subunit vaccine in mice, by enhancing antigen-specific binding antibody responses, improving neutralizing antibody titers and kinetics, and increasing IFN-γ-associated cellular immunity. These findings support PCP-II as a promising natural adjuvant candidate for SARS-CoV-2 subunit vaccines.

## 5. Conclusions

PCP-II enhanced humoral immunogenicity, improved neutralizing antibody responses, and was associated with increased IFN-γ-related cellular responses in an RBD subunit vaccine model in mice. In this comparative evaluation system, PCP-II emerged as the most active polysaccharide fraction among those tested, supporting its potential as a candidate adjuvant for protein subunit vaccines.

## Data Availability

The data presented in this study are available in the article and Supplementary Materials.

## Supplementary Materials

The following supporting information can be downloaded at: https://www.mdpi.com/article/10.3390/vaccines14050389/s1, The original data of all figures.
