# Supplementary material for: Poria cocos Polysaccharide Fraction PCP-II Enhances Humoral and Cellular Responses to a SARS-CoV-2 RBD Subunit Vaccine in Mice
Source: Vaccines (Basel). 2026 Apr 27;14(5):389. doi: 10.3390/vaccines14050389 (PMC13211347; doi:10.3390/vaccines14050389)
Supplement: Supplementary file 1 [file vaccines-14-00389-s001.zip › vaccines-4224899-supplementary.pdf]

The original data of Figure 1

ODs for total IgG

Group	Control	RBD	PCP-I+RBD	PCP-II+RBD	IIP+RBD	Alhydrogel+RBD
	0.27	0.21	1.22	1.79	0.22	0.77
	0.36	0.45	0.23	1.64	0.44	0.81
	0.24	0.34	0.18	3.11	2.51	0.71
		1.41	0.51	1.47	0.69	1.01
		0.61	2.65	2.49	0.58	1.02
		0.30	0.32		0.44	0.45
			1.20		0.87	0.23

ODs for total IgG1

Group	Control	RBD	PCP-I+RBD	PCP-II+RBD	IIP+RBD	Alhydrogel+RBD
	0.98	1.29	1.13		1.83	5.13
	0.94			4.90	1.98	
	0.96		1.23	5.06	1.90	2.31
		1.24		18.51		2.76
		2.76	1.39		1.39	2.98
		1.20	2.90			1.98
			4.25		1.31	

ODs for total IgG2a

Group	Control	RBD	PCP-I+RBD	PCP-II+RBD	IIP+RBD	Alhydrogel+RBD
	0.90		0.86	0.91		
	0.87	0.91	1.05	0.89	0.92	0.84
	1.04		0.84	0.88	0.95	0.89
		0.80	0.99		0.90	0.99
		0.98				0.96
		0.80	0.92		0.89	
					0.93	0.87

ODs for total IgG2b

Group	Control	RBD	PCP-I+RBD	PCP-II+RBD	IIP+RBD	Alhydrogel+RBD
	0.93	1.31	2.07	1.35	1.13	1.93
	0.74	1.67	1.80	2.02	1.57	0.89

	0.79	0.95	1.09	1.19	1.00	1.07
		0.95	0.94	1.12	1.21	0.98
		1.72	0.86	1.03	0.92	1.09
		0.82	1.08		0.85	0.77
			1.05		0.85	0.99

ODs for total IgG2a/IgG1

Group	Control	RBD	PCP-I+RBD	PCP-II+RBD	IIP+RBD	Alhydrogel+RBD
	0.92		0.76			
	0.93			0.18	0.46	
	1.08		0.68	0.17	0.50	0.39
		0.65				0.36
		0.36				0.32
		0.67	0.32			
					0.71	

Places where data is not filled in indicate missing data.

The original data of Figure 2

Neutralizing Antibody titer(1:X)

Group				IIR+RBD			
Day14	8		8	0		64	8
Day28	80	40	160	160	128		
Day56	80	40	160	80	80		
Group				PCPI+RBD			
Day14	0	0	0	0	0		
Day28	0	0	0	0	0		
Day56		0	0	0	10	10	
Group				PCPII+RBD			
Day14		0	0	0		8	
Day28		64		64	256	16	
Day56	160		80		640	80	
Group				AL+RBD			
Day14	128	128	128	128		32	
Day28	256	128			256	256	128
Day56	640	640		640		1280	1280
Group				RBD			
Day14	0	0	0	0	0		
Day28	16		16	32	64	16	
Day56	160		20	160		20	

Group	control						
Day14	0	0	0	0	0	0	0
Day28	0	0	0	0	0	0	0
Day56	0	0	0	0	0	0	0

Places where data is not filled in indicate missing data.

The original data of Figure 3

B cells% of lymphocytes（The result repeated twice）

Group	RBD	PCP-I+RBD	PCP-II+RBD	IIP+RBD	Alhydrogel+RBD
B cells% of lymphocytes	47.7	42.9	34.5	37.6	36
B cells% of lymphocytes	32	42.2	53.4	34.2	50

The original data of Figure 4

SFCs/2.5×106Splenocytes(IFN-γ)

Group	Control	RBD	PCP-I+RBD	PCP-II+RBD	IIP+RBD	Alhydrogel+RBD
	2	4	57	40	13	8
	2	8	59	48	18	12
	2	3	72	40	7	6
	1	4	59	42	29	10
	3	5	79	24	2	4
	2	2	58	61	13	2

SFCs/2.5×106Splenocytes(IL-4)

Group	Control	RBD	PCP-I+RBD	PCP-II+RBD	IIP+RBD	Alhydrogel+RBD
	33	337	512	386	242	344
	32	520	583	604	135	547
	21	363	519	424	244	484
	31	511	606	662	73	569
	23	347	514	435	258	448
	32	474	537	629	67	600
